# Cell Microarray Technologies for High-Throughput Cell-Based Biosensors

**DOI:** 10.3390/s17061293

**Published:** 2017-06-05

**Authors:** Hye Jin Hong, Woong Sub Koom, Won-Gun Koh

**Affiliations:** 1 Department of Chemical and Biomolecular Engineering, Yonsei University, 50 Yonsei-ro, Seodaemun-gu, Seoul 120-749, Korea; ellenhong91@yonsei.ac.kr; 2 Department of Radiation Oncology, Yonsei University College of Medicine, 50 Yonsei-ro, Seodaemun-gu, Seoul 120-749, Korea; mdgold@yuhs.ac

**Keywords:** cell-based biosensor, cell microarray, microfabrication, toxicology, drug discovery, biosensing, positional array, suspension array

## Abstract

Due to the recent demand for high-throughput cellular assays, a lot of efforts have been made on miniaturization of cell-based biosensors by preparing cell microarrays. Various microfabrication technologies have been used to generate cell microarrays, where cells of different phenotypes are immobilized either on a flat substrate (positional array) or on particles (solution or suspension array) to achieve multiplexed and high-throughput cell-based biosensing. After introducing the fabrication methods for preparation of the positional and suspension cell microarrays, this review discusses the applications of the cell microarray including toxicology, drug discovery and detection of toxic agents.

## 1. Introduction

A biosensor is a bioanalytical device or system in which biomolecules such as nucleic acids, proteins (enzymes or antibodies), and whole cells are used as the recognition elements. Biosensors allow researchers to indirectly measure target analytes using biological receptors via well-understood transducers. Although biosensor studies have primarily been focused on diabetes, they have potential uses in numerous other areas such as drug screening, detection of chemical warfare agents, environmental toxins, and pollutants; and monitoring of food safety [1,2].

Among various types of biosensor, cell-based biosensor that use living cells as the sensing element have been developed rapidly for various applications. The key advantage of cell-based assay over conventional bioassay based on nucleic acids and proteins is that it can provide functional information as well as analytical information, where the former is the information about the physiological effect of analytes on our bodies, and the latter is the information about how much of a specific substance is present [3,4]. Although most of the cell-based assays are currently being carried out using well-plate formats, a lot of efforts have been made to enhance the performance of cell-based assay systems by means of miniaturization [5,6]. In order to miniaturize the cell-based assay system, cell microarray have been fabricated via different microfabrication techniques, where thousands of cells are attached either on a flat surface (planar or positional array) or on a particle (solution or suspension array) to implement multiplex assays in a high-throughput manner [7,8,9,10,11]. Cell microarrays are eventually combined with optical or electrical detection technologies and integrated into microfluidic systems to monitor the changes in cellular behavior under the influence of the external environment.

In this article, we first give detailed introduction about the types of cellular microarrays that are prepared with planar substrates, microparticles, or a biomimetic three-dimensional (3D) environment. Then, we discuss the applications of cell microarrays such as drug screening and biosensor systems.

## 2. Preparation of Cell Microarray

**Figure 1:**
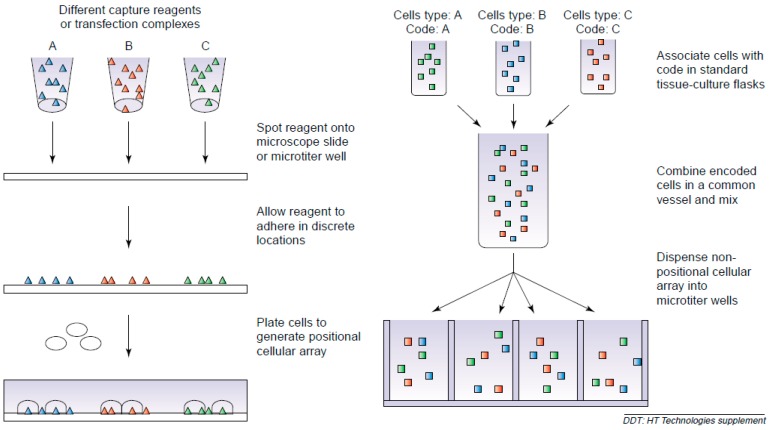
Schematic representation of positional array and suspension microarray (reproduced with permission from [12]).

In cell microarrays, different types of cells can adhere and grow either on flat chip-like substrates or on particles to implement multiplexed and high-throughput cell-based assays, where former is called as positional arrays and the latter is suspension arrays (Figure 1) [12].

### 2.1. Positional Arrays

Positional arrays are usually prepared by generating multiple cellular microspots on the planar substrates. For example, a positional array can contain numerous spots of cells on one glass slide, and each spot is easily distinguished by (*x*, *y*) coordinates on the microarray. Positional cell microarrays were initially prepared by physical spotting of cells on substrates [13,14]. Nonetheless, due to recent advances in microelectromechanical systems (MEMS) and their applications to biology, surface-micropatterning processes such as photo- and soft lithography are widely used to generate cell microarrays on various substrates [15,16].

**Figure 2:**
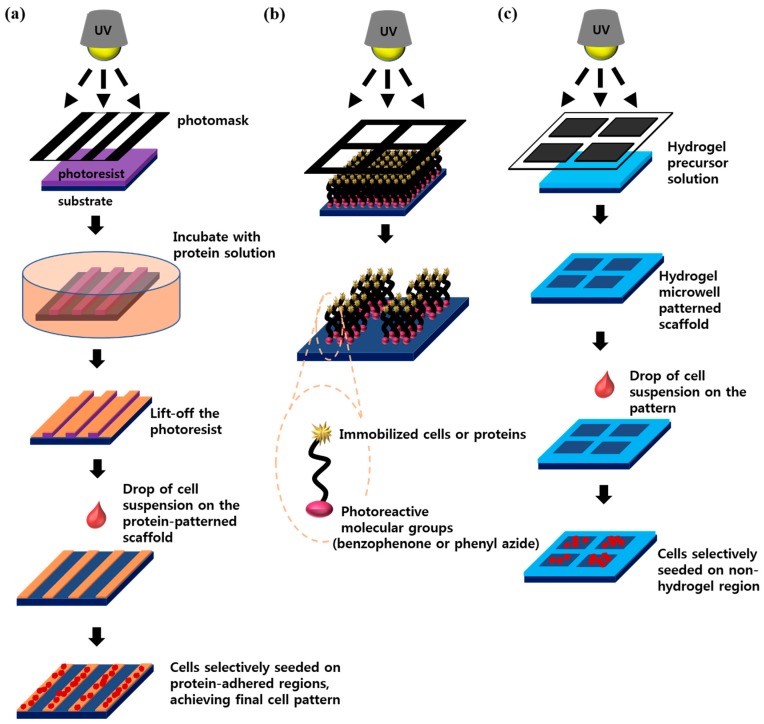
Cell and protein patterning using photolithography: (**a**) Lift-off approach using photoresist template; (**b**) Photochemical fixation of biomolecules; (**c**) Using photopatterned hydrogel microstructures.

Photolithography has been most extensively used for cell patterning due to its simple way of producing such patterns. This review describes three ways to generate cellular micropatterns using photolithography. First, a cell microarray can be prepared by photopatterning and a lift-off process, where the generated micropatterns act as templates as shown in Figure 2a. For example, a designed micropattern was generated from the photoresist after ultraviolet (UV) light exposure through the photomask and developing process. Then, the micropatterned substrate was coated with cell-adhesive proteins [17,18]. Finally, by lifting off the photoresist and incubating the substrate with a cell solution, technologist can obtain the desired pattern of cells [19,20]. The second method represent the use of photoreactive groups such as phenyl azide and benzophenone as shown in Figure 2b [21,22]. In this method, a substrate is covered with photoreactive molecules conjugated with cell-adhesion proteins. A photopatterning process causes the covalent bonding between substrates and photoreactive groups only at UV-exposed areas. After removal of any unreacted molecules with a solvent and subsequent cell seeding, the desired cellular micropatterns are obtained [10,23,24]. The final method is the use of photo-crosslinkable hydrogel micropatterns based on the fact that hydrogels are generally non-adhesive to proteins and cells [25,26]. The previous studies showed the fabrication of hydrogel micropatterns using poly(ethylene glycol)-diacrylate (PEG-DA) on various substrates. When microwell-type hydrogel micropatterns were fabricated, cells selectively adhered to the hydrogel-free region as shown in Figure 2c [27,28]. Besides the PEG hydrogel, other hydrogels such as those based on hyaluronic acid and gelatin have been photopatterned and used to create cellular micropatterns [29,30,31].

**Figure 3:**
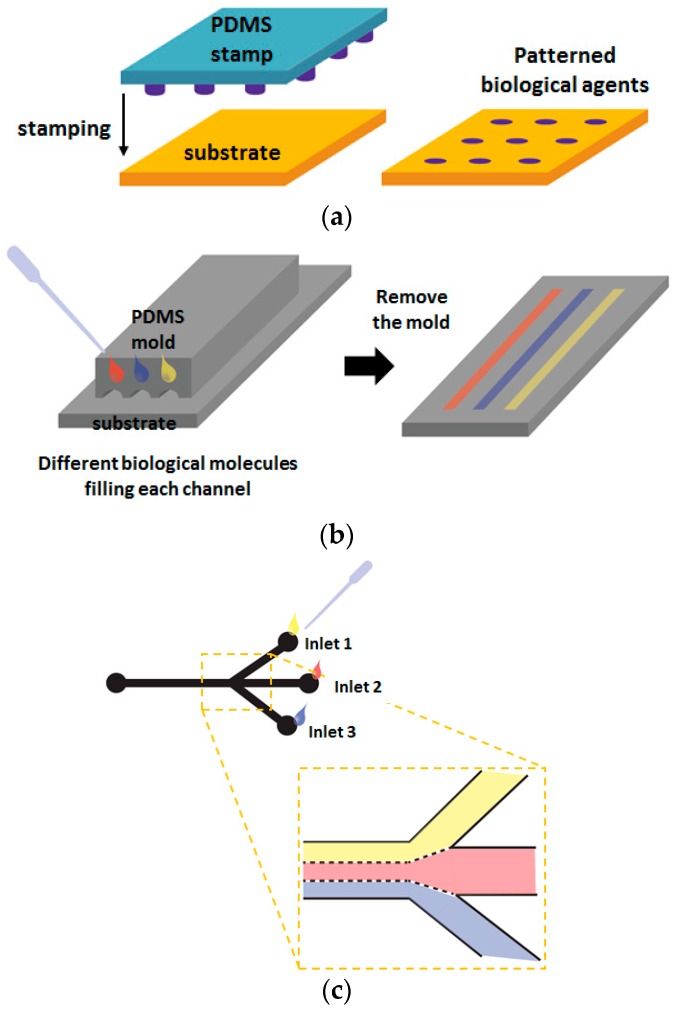
Schematic illustration of soft lithographic techniques for micropatterning: (**a**) Microcontact printing; (**b**) Microfluidic patterning; (**c**) Laminar flow patterning [11].

Soft lithography, which involves soft elastomeric materials for pattern transfer, was developed by Whitesides and colleagues as an alternative to photolithography because soft lithography is more suitable for biological applications [11,32]. Currently, soft lithographic techniques such as microcontact printing (μ-CP) and microfluidic channels involving poly(dimethylsiloxane) (PDMS) are mostly used for cell patterning [33,34]. In the μ-CP method, micropatterned PDMS act as stamp that transfer the patterns of certain molecules onto the substrate. (Figure 3a) [35], and this method represents great producibility due to re-usable stamp unless it is not physically damaged. A number of the studies on the cell and protein patterning using μ-CP have involved the use of alkanethiols, HS(CH_2_)_n_X, which chemisorb on metal surfaces such as gold, and form self-assembly monolayers (SAMs) [36,37,38]. The capability to have various terminal groups (X) enables the alkanethiols to control the surface properties. If SAMs were terminated with oligo(ethylene glycol) or ethylene glycol, those surfaces become non-adhesive to cells and proteins [39,40]. The combination of μ-CP with alkanethiol-based SAM can generate cell adhesive and non-adhesive microdomains on metal surfaces, which can control the position of cells and generate cellular micropatterns. The PDMS-based microchannels are also used as a simple method for patterning various biological molecules [41,42] since each microchannels can be filled with different solutions. Microchannels were formed by reversible sealing of micropatterned PDMS with a substrate. When a small volume solution is dropped on the substrate near the channel entrance, microchannels are filled with solutions via capillary forces (Figure 3b) and only microliters of the solution is necessary to fill the microchannels [43,44,45]. Microchannels have been used to generate cellular micropatterns on the various substrates. Here, cell adhesive protein patterns were firstly created on surfaces by introducing cell-adhesion proteins such as fibronectin and collagen into the microchannels [46,47]. After removal of PDMS molds, patterned substrates are seeded with cells so that the cells adhere only to the patterned proteins. By introducing various cell types through different channels, a micropattern consisting of different cell types may also be easily generated on surfaces [48,49,50], allowing multiphenotypic observation on a single plane.

Due to the small dimension of microfluidic systems, the solution inside microchannels moves as a laminar flow [51,52,53]. Therefore, when the solutions introduced from different inlet are merged into a single microchannel, each solution flows parallel to each other without mixing with turbulence, which can be used to generate micropatterns as shown in Figure 3c, for example, if each solution contains different proteins [54,55].

**Figure 4:**
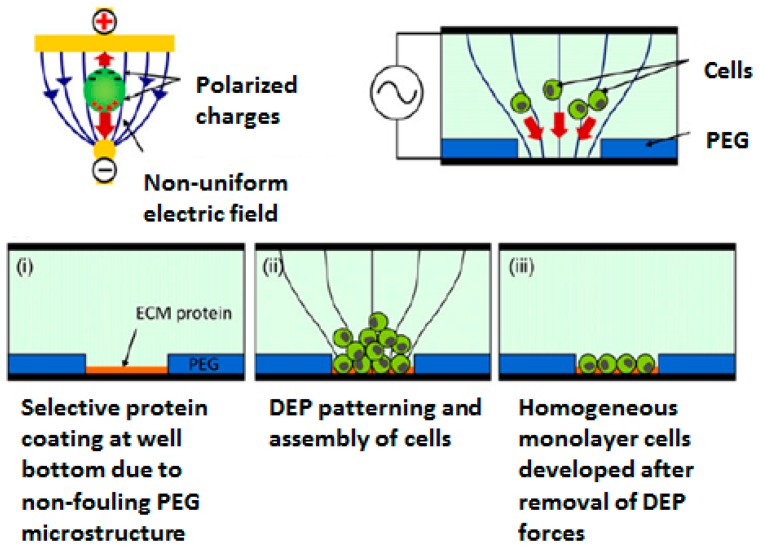
Example of generating cellular micropatterns using dielectrophoresis (DEP) (reproduced with permission from [62]).

In addition to photo- and soft lithography, which passively direct cell attachment by means of cell adhesive and non-adhesive microdomains, cellular micropatterns can be generated by manipulation techniques where cells are positioned to specific location by applying external forces [56,57]. Although various governing forces can be used to trap and localize cells, creating cellular micropatterns using dielectrophoresis (DEP) have received much attention. The cell patterning using DEP has extensive advantages such as the absence of the cell pretreatment, good cyto-compatibility, large-scale parallel functioning, high spatial resolution, and easy combination with other techniques, thus endowing the excellence of DEP in handling a lot of living cells at a time [58,59,60,61]. For example, Tsutsui et al. applied DEP to cell patterning. DEP causes a motion of polarized cells through non-uniform electric fields, and by means of patterned microelectrode arrays, more purified configuration can be obtained (Figure 4) [62].

### 2.2. Suspension Arrays

**Figure 5:**
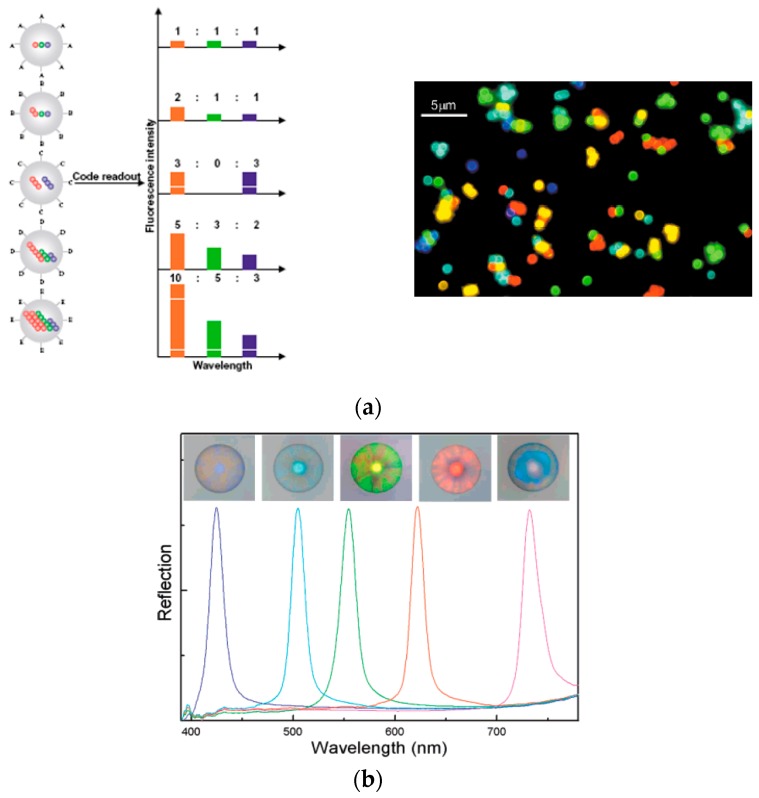
Optically encoded microparticles: (**a**) QD-incorporated microbeads; (**b**) Silica photonic crystal microspheres (reproduced with permission from [74,77]).

Although positional arrays have been well established and widely applied for high-throughput assays, suspension arrays are rising as an alternative microarray format because it is believed that they have greater flexibility than positional arrays in terms of multiplexity [63,64]. In the case of positional microarrays, patterning of different cells on stand-alone substrates is difficult and requires a complex process. However, with suspension arrays, multiplex assay systems can be easily achieved by simply mixing the independently prepared microparticles carrying different cells. In this case, the identity of the cells is determined by self-encoded microparticles that contain the cells as array component, instead of *x*-*y* coordination on a planar surface [65,66,67]. Although a variety of strategies has been investigated to encode microparticles, e.g., optical, electronic, photophysical, and graphical encoding, optically coded spherical microparticles are most commonly employed to realize the multiplexed assays in suspension array formats [68,69,70,71,72]. For discriminatory optical detection, mainly two types of encoding elements are incorporated into microparticles: fluorescent dyes and quantum dots (QDs). The latter has become alternative probes for suspension arrays instead of usual fluorescent dyes owing to a wide excitation wavelength, their high quantum yield, and excellent photostability compared with fluorescent dyes [73]. Figure 5a shows that the different color of QDs are embedded into microparticles with various ratios to identify each particle [74]. However, there is still a possible disadvantage of QDs as a source of optical fluorescence because of their toxicity. To avoid this problem, Zhao et al. and Deng et al. have produced silica colloidal crystal beads (SCCBs) and silica photonic crystal microspheres (SPCM) as carriers for the suspension array (Figure 5b) [75,76]. Their end products generally share the common ideas: e.g., both groups have used silica nanoparticles as the fundamental material for microspheres. The coding for these beads is a reflection of their own structural periodicity, so they could avoid bleaching and quenching of optical intensity, whereas chemical instability is reduced.

**Figure 6:**
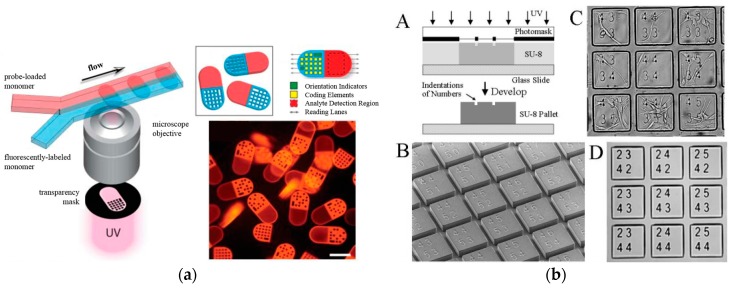

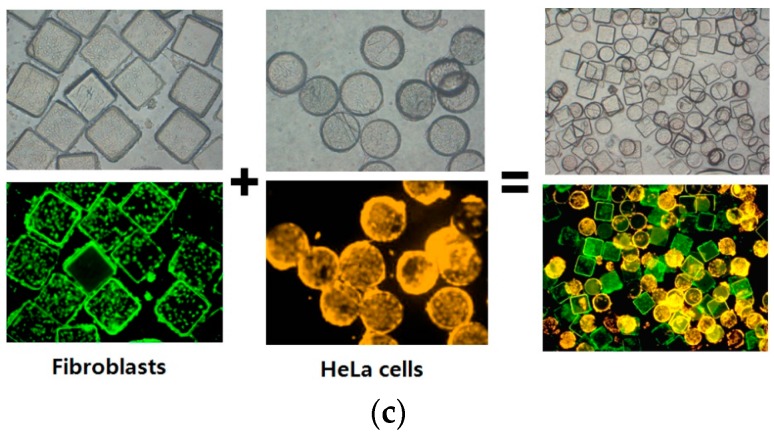
Graphically or shape-coded microarray: (**a**) Schematic diagram of the synthesis of bar-coded hydrogel microparticles using flow lithography; (**b**) Fabrication of number-encoded micropallet array with fibroblasts cultured on the surface of the array; (**c**) A suspension microarray of microboards that contained multiple cell types (fibroblasts and HeLa cells), where each cell was identified by shape of microboards (reproduced with permission from [83,85,87]).

However, there are several disadvantages of using optical encoding method. First, the number of color combination that can be generated is very limited. Second, there is a possibility for encoding color to be overlapped with colors used for the target detection or cell staining. Because of those drawbacks of optical encoding method, graphically or shape-coded microparticles were proposed as new formats for suspension arrays [78,79,80]. Doyle’s group invented continuous and stop flow lithography, which are capable of fabricating different shapes of microparticles [81,82]. For example, bar-coded microparticles divided into coding and detecting microdomains were prepared as shown in Figure 6a [83]. Albritton and Koh’s group developed a suspension cell microarray using the SU-8 micropallet (or microraft) and a microboard, where each cell was identified by the barcode on the SU-8 micropallet or by shapes of SU-8 microboards, respectively (Figure 6b,c) [84,85,86].

## 3. Cell Microarrays in a Biomimetic Environment

**Figure 7:**
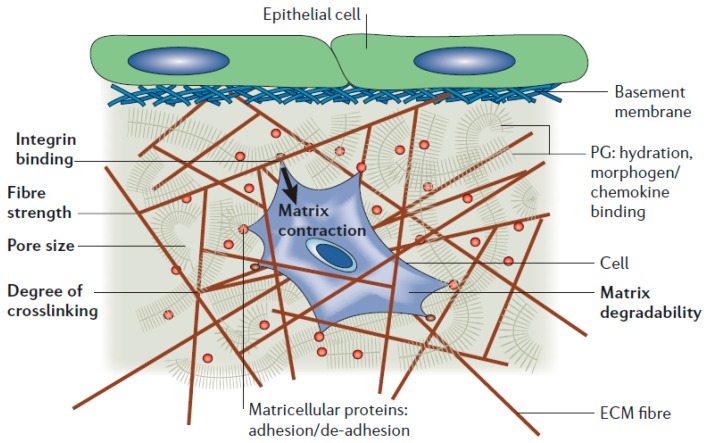
Three-dimensional environments for cells in vivo (reproduced with permission from [89]).

In most cases of cell microarray preparation, cells are manipulated to adhere to a two-dimensional (2D) substrate for both the positional and suspension array system. In a real in vivo environment, cells are present in a 3D extracellular matrix (ECM) composed of a nanofibrous network whose interfibrous space is filled with hydrogel-like materials consisting of proteins and polysaccharides as shown in Figure 7 [88,89].

Therefore, in 2D system, cells exist in an unnatural environment and therefore, the cellular responses to external stimuli in a 2D microarray system might be different from those of cells in real tissues [90,91,92]. In order to minimize the difference between a cell-based assay and an animal study, there have been many efforts to create cell microarray in a biomimetic environment. One way to overcome the problems related with a 2D culture is to conduct cellular experiments in a biomimetic 3D culture system, which has been mostly achieved by means of a hydrogel and nanofiber-based matrix [93,94].

### 3.1. Hydrogel-Based 3D Cell Microarrays

**Figure 8:**
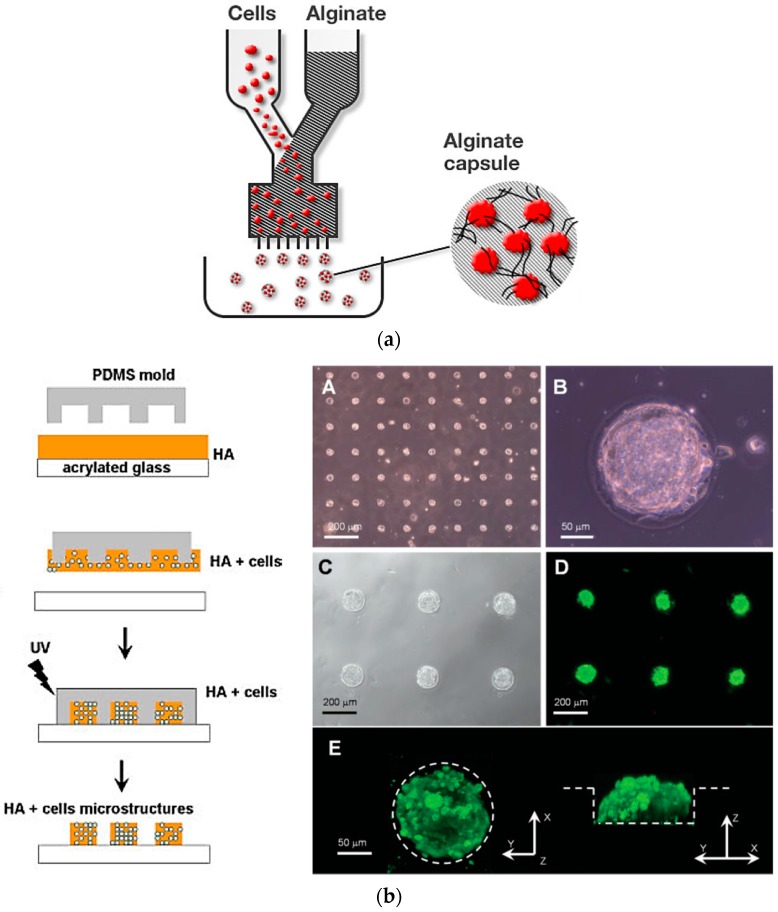
Encapsulation of cells within hydrogel microstructures: (**a**) Schematic illustration of preparing cell-entrapping alginate microbeads; (**b**) Microarray of cell-encapsulating HA hydrogel prepared by combining micromolding and photocrosslinking (reproduced with permission from [105,107]).

Among many types of biomaterials that have been fabricated to mimic ECM, hydrogel has become one of the excellent candidates for the particular purpose. With the emerging concept of 3D microarray systems, hydrogels have been used as a novel platform for cellular microarray applications. A hydrogel is a 3D hydrophilic crosslinked network made from water-soluble polymers. When placed in an aqueous solution, they tend to swell and retain a substantial amount of water within [95,96,97]. Hydrogels have been widely used as biomaterials because of their high water content, softness, pliability, biocompatibility, and easily controlled mass transfer properties that are essential for allowing transport of nutrients to (and waste products from) the cell [98,99]. A hydrogel-based 3D cell microarray is prepared by encapsulating mammalian cells inside a hydrogel microarray to mimic the in vivo environment so that a more accurate response of cells to analytes can be obtained [100,101]. Cell encapsulation strategies usually involve homogenization of cells with gel precursors in a liquid state followed by gelation through a crosslinking procedure [102,103]. Sugiura et al. fabricated cell-encapsulating alginate hydrogel microbeads. Alginates are anionic polysaccharides and can form hydrogels in the presence of multivalent cations such as Ca^2+^ (Figure 8a). Cells are resuspended in a sodium alginate solution, and the droplets of the cell-containing alginate solution are injected into a CaCl_2_ solution using a microinjection system to form hydrogel microbeads encapsulating cells [104,105,106]. The Khademhosseini group used micromolding technique to generate cell-encapsulating hyaluronic acid (HA) hydrogel microstructures (Figure 8b). Here, an acrylated HA solution containing a cell suspension was exposed to UV light and became crosslinked, thus encapsulating cells [107].

**Figure 9:**
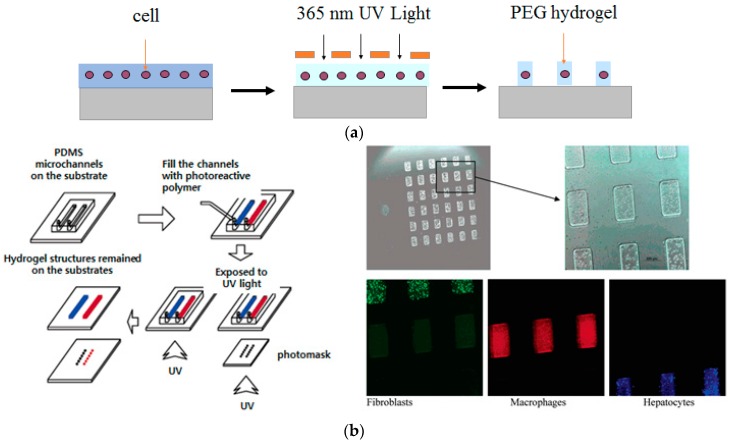
PEG-based hydrogel microarray encapsulating mammalian cells: (**a**) Scheme of preparing cell-encapsulating PEG hydrogel micropatterns by photolithography; (**b**) Fabrication of array of hydrogel microstructure containing three phenotypes of cells using photoreaction injection molding (reproduced with permission from [54,113]).

PEG-based hydrogels have also been widely used to create 3D cell microarrays. The physical properties of PEG-based hydrogels can be easily controlled by varying the molecular weight of the PEG molecules, and the transparent nature of PEG hydrogels also makes them suitable for various detection schemes in biosensing applications [108,109,110]. Cell-encapsulating PEG microarrays can be prepared by simple photolithography (Figure 9a) or via photoreaction injection molding (Figure 9b) [111]. In particular, photoreaction injection molding offers several advantages over the previously described methods of encapsulating mammalian cells in hydrogel microstructures. For example, a small volume of a cell-containing precursor solution is sufficient to fill and to photopolymerize inside a microchannel, whereas cell patterning techniques based on spin-coating require a much larger volume of the precursor solution because of a solution loss during the spin-coating procedure [112]. Another important advantage of photoreaction injection molding is the possibility to encapsulate different phenotypes on the same array as shown in Figure 9b because each microchannel can be independently filled with a hydrogel precursor solution containing different cell types [113]. Because most of the hydrogels do not support cell adhesion and growth, cell adhesion proteins or peptides were incorporated into a hydrogel when cells were encapsulated within the hydrogels [114]. Although such cell-encapsulating process could lead to substantial cellular damages, highly-adaptable in vivo condition of cells that hydrogels provide is still inevitably appealing. Cell-encapsulating hydrogel microarrays were also fabricated within microfluidic devices to realize lab-on-a-chip systems [115]. Microfluidic systems offer several advantages, including decreased sample volume, fewer cells, shorter reaction time and the ability to perform many experiments in parallel. Microfluidic devices are well suited for biological experiments at the cellular level because microchannels within these devices can mimic the physical size observed in vivo. Because of the small size of microchannels, microfluidic devices also enable adequate oxygenation and fast nutrition diffusion. Such an environment helps cells to easily maintain a local microenvironment (in contrast to macro-scale cell culture flasks) and to exist in a less stressful, more in vivo-like surroundings, which can lead to more accurate data on cellular behavior in response to external stimuli [116,117,118,119,120].

While most of previous studies have discussed homogeneous network of hydrogels driven from a single type of material, the hydrogel features can be advanced if two or more types of polymeric materials are combined. This kind of hydrogels, so called the hybrid hydrogels, can take various forms depending on the materials used, and usually these forms are achieved by combinations of natural polymers and synthetic polymers, for example, alginate-poly-l-lysine(PLL), alginate-acrylate, collagen-alginate, etc. [121]. Vlassak et al. has developed hybrid hydrogels with increased stiffness and toughness for cartilage regeneration by combining alginate and polyacrylamide when forming hydrogels [122]. Yarmush et al. has formed microencapsulation system with alginate-PLL hydrogels to satisfy both cell viability and sufficient stiffness while supporting stem cell differentiation [123].

### 3.2. Nanofiber-Based Microarrays

Electrospun polymeric nanofiber scaffolds that have high porosity and a large surface area are attractive substrates for cell patterning. Nanofiber can recapitulate characteristics of native cellular microenvironments for in vitro cell cultures. Various cell types have been seeded onto electrospun fibers, with the results indicating that fibrous structures have a more favorable influence on cell growth than planar 2D surfaces do [124,125,126,127,128]. Although a lot of nanofiber-related studies have been reported, most of them involve control of the properties of either the nanofiber itself (nanometer scale) or of the macroscopic nanofiber matrix (millimeter or centimeter scale) [129,130].

**Figure 10:**
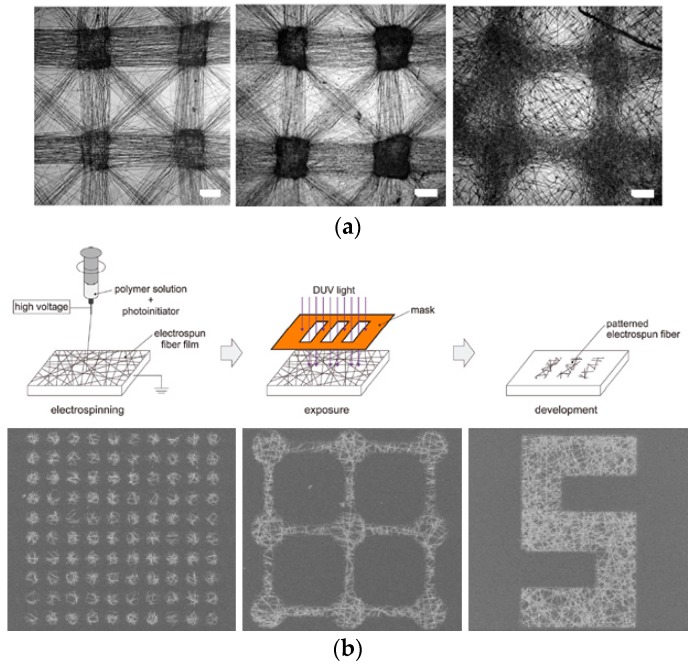

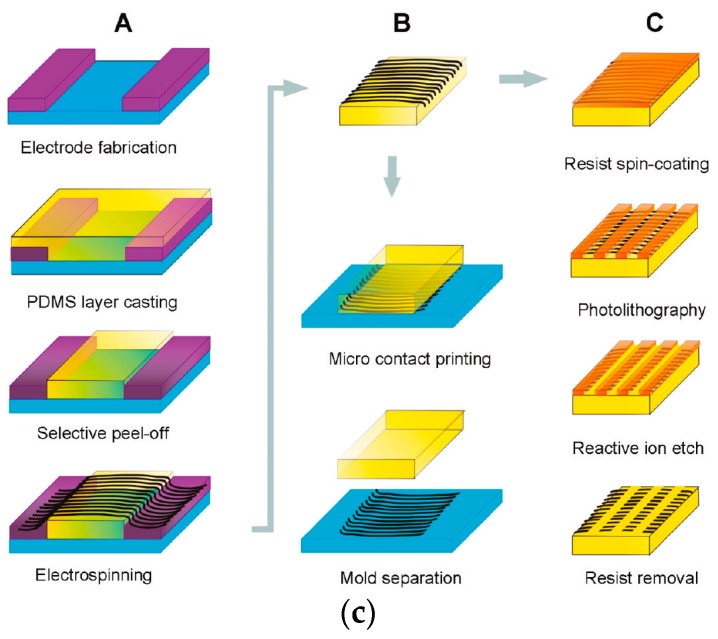
Preparation of micropatterned nanofiber: (**a**) Selective deposition of nanofiber using the micropatterned electrodes; (**b**) Fabrication of micropatterned nanofiber using photopatterning; (**c**) Schematic process flow of preparing nanofiber micropatterns via microcontact printing of fibers deposited on a PDMS stamp and lithographic patterning of fibers on a PDMS stamp by using photolithography and reactive ion etching techniques (reproduced with permission from [131,132,135]).

Despite the extensive research that has been conducted on nanofiber and macroscopic configurations of a nanofibrous matrix, only a few studies have shown implementation of micropatterning electrospun nanofibers. For example, selective deposition of nonwoven mats using collectors with microscopic electrode patterns may achieve dense nanofiber deposition within specific microdomain (Figure 10a). Furthermore, electrospinning of photoreactive polymers and subsequent photopatterning can generate spatially well-defined fibrous micropatterns (Figure 10b), whereas multistep microcontact printing and etching techniques may be used to micropattern electrospun fibers (Figure 10c) [131,132,133,134].

**Figure 11:**
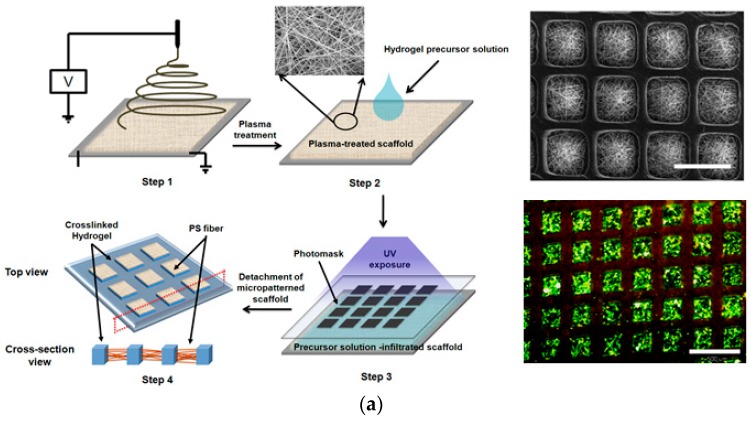

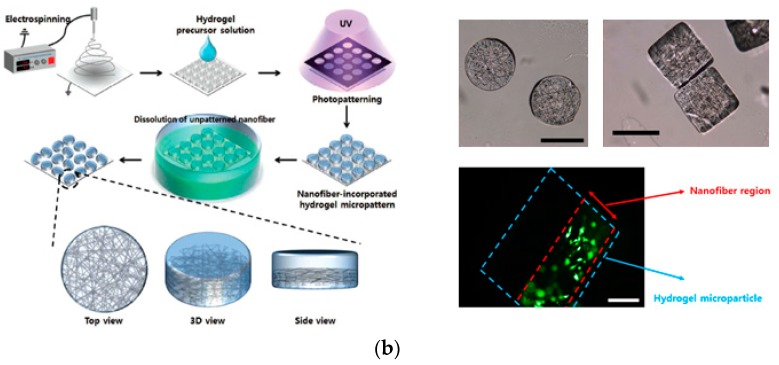
Creation of cellular micropatterns using hydrogel-incorporated nanofiber matrix: (**a**) Schematic diagram of preparing micropatterned fibrous scaffold by combining PEG hydrogel lithography with electrospinning technique (left), and SEM image of resultant scaffold and fluorescence image of cellular microarray (right); (**b**) Schematic illustration of preparing hydrogel microparticles entrapping nanofibers (left), and optical images of different shapes of hydrogel microparticles entrapping PCL nanofiber and side view of hydrogel microparticles encapsulating nanofiber-attached fibroblasts (right) (reproduced with permission from [136,137,139]).

Recently, Koh and colleagues fabricated positional and suspension types of cell microarrays using a micropatterned nanofiber matrix which was prepared by combining PEG hydrogel lithography and electrospinning (Figure 11a) [136]. For the positional cell microarray, microwell-shaped hydrogel micropatterns were incorporated into nanofiber as shown in SEM image of Figure 11a [137]. Owing to the non-adhesiveness of PEG hydrogels, cells selectively adhered onto the nanofiber region, creating cellular micropatterns in a 3D environment (fluorescence image of Figure 11a) [138]. For a suspension cell microarray, a micropillar-shaped PEG hydrogel precursor solution was photopatterned in the presence of nanofiber. Subsequent removal of the bare nanofiber yielded various shapes of nanofiber-entrapped hydrogel microparticles as shown in Figure 11b [139,140]. When a cell suspension was added to the precursor solution, the cells were encapsulated within hydrogel microparticles during the crosslinking process and adhered onto nanofiber because of non-adhesiveness of the PEG hydrogel (fluorescence image of Figure 11b) [141].

Nanofibers may be incorporated into microfluidic devices. Jiang et al. have proposed a facile nanofibrous scaffolds arrayed with microfluidic channels, expanding the practicality of electrospun nanofibers. A biocompatible nanofibrous sheet was electrospun on a glass slide with an array of holes, and then a photoresist was cast on top followed by UV exposure. The resulting scaffold satisfied 3D cellular growth on the microarrayed platform [142]. The difficulty with production of a nanofiber matrix of uniform size and thickness and with handling small and thin nanofiber matrices may be overcome by incorporating hydrogel micropatterns. The resultant hydrogel-framed nanofiber may be integrated into microfluidic devices [143]. In addition to conventional 3D cell culture using hydrogel or nanofibers, newly emerged 3D surface based on supramolecules, copolymers and porous silicon has recently received great attention as a new substrate for 3D cell microarrays [144,145,146]. So far, we introduced different methods to prepare cell microarray in 2D or 3D environments and Table 1 lists the advantages and disadvantages of each method.

## 4. Applications of Cell Microarrays

Cell-based microarrays are powerful analysis tools for high-throughput testing of many target samples. Miniaturization allows the increase of assay throughput, reduction of reagent consumption and the number of cells required, thus making microarray system attractive for a variety of analysis such as drug discovery, biosensing, and toxicology. In those applications, cells in microarrays are exposed to samples containing drugs, pathogens, pollutants and various biomolecules. Cellular response against those external stimuli can be monitored through optical (fluorescence or absorbance) or electrical (change of impedance or electrical potential) detection methods [3,147].

### 4.1. Toxicology

Evaluating the toxicology has been recognized as the important study since we were getting more knowledge about the toxicity, harmfulness and the resultant adverse effect of various compounds. In the field of toxicology, in vivo work using animal has an advantage over in vitro work in that it takes into account the response of entire biological system to a chemical challenge. However, use of living animals not only is expensive and cumbersome but also has ethical issue. Cell-based assay was proposed as an alternative method to evaluate the potential toxicity of certain molecules [148]. Like other application, cell microarrays are necessary for high-throughput toxicity assay [149].

**Figure 12:**
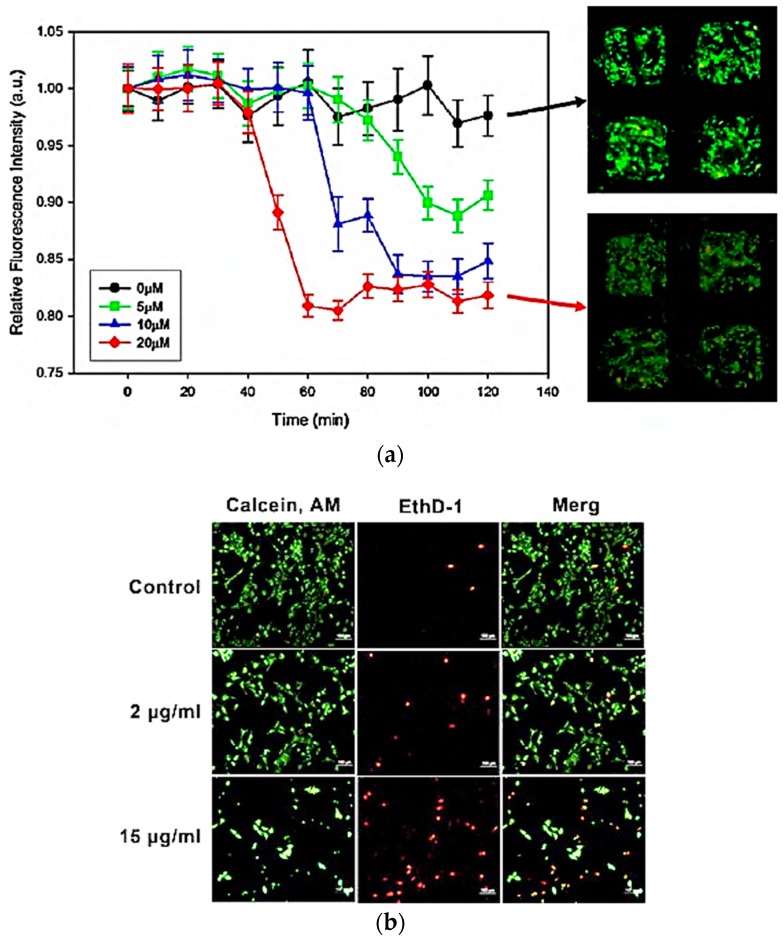
Change in fluorescence intensity representing cell viability: (**a**) Fluorescence image and graphical data of fibroblasts survival depending on various concentration of sodium azide; (**b**) Observation of cellular viability depending on various concentration of silver nanoparticles (AgNPs) feed to NIH 3T3 cells. (reproduced with permission from [139,158]).

Toxicity assay is being mostly carried out by quantifying viable and dead cells within the microarray after cells are exposed to environmental perturbation caused by target compounds. The changes of optical and electrical signal induced by the decrease of viable cells are detected in cell-based toxicity studies, which are classified into two methods depending on whether they are label-based or label-free assay [149,150]. Label-based assays use colorimetric or fluorescent viability/cytotoxicity assay to quantify the portion of living and dead cells for toxicity assessment, where the changes of color and fluorescent signals from the cells before and after exposure to a certain compound result from the difference in cell membrane permeability or metabolic activity between living and dead cells. The MTT assay, which is most commonly used as viability assay, is an example of colorimetric assay measuring cell metabolic activity. In this assay, MTT (3-(4,5-dimethylthiazol-2-yl)-2,5-diphenyltetrazolium bromide) is reduced to purple formazan by the enzymes within the cells. The reduction of MTT by intercellular enzyme only occurs within the living cells, and dead cells cannot cause this change [151,152]. Therefore, the absorbance of solubilized formazan is proportional to the number of cells. However, MTT assay cannot be applied to cell microarray system since it measures the absorbance of supernatant containing dissolved formazan. Another type of metabolic activity assay uses the fluorescent reporter such as fluorescein diacetate and calcein AM, which are initially colorless and non-fluorescent molecules but converted into fluorescent molecules such as fluorescein and calcein, respectively once they are hydrolyzed by esterase enzymes inside cells [153,154]. Dead cells cannot carry out this conversion and consequently, fluorescence intensity from resultant fluorescein or calcein are related with the viability of cells. Figure 12a shows that green fluorescence intensity from calcein decreased when cells in microarrays were exposed to the toxic molecules [139]. Since produced fluorophores are trapped inside the cell, this assay is applicable to cell microarray by allowing in-situ monitoring of fluorescence intensity from the cells in each microarray spot.

In the case of viability assays based on membrane permeability, trypan blue or naphthalene black are used as colorimetric reporters, which are originally membrane-impermeable dye but can permeate into impaired cell membrane of dead cells and stain cytoplasm of dead cells [155]. Fluorescence-based assays can be also used by addressing membrane integrity [156,157]. One example is a Live/Dead viability/cytotoxicity fluorescence assay that uses SYTO10 and Dead Red as fluorophores to distinguish living cells and dead cells. SYTO 10 stains living cells green and Dead Red stains dead cells red based on the difference in the membrane integrity between living cells and dead cells as shown in Figure 12b [158].

On the other hands, lactate dehydrogenase (LDH) assay detects the LDH activity released from the cells with damaged membranes [159,160]. Since this method measures the fluorescence or absorbance of supernatant in extracellular space, it cannot be easily incorporated with cell microarray like MTT assay.

Label-free assays usually monitor the changes in cell shape and morphology induced by external stimuli such as toxin or drugs. Various microscopic techniques were proven to be suitable to carry out phenotypic screening of cells on cell microarrays. Non-imaging tools such as surface plasmon resonance (SPR) or resonant waveguide grating also demonstrated their capability to monitor stimuli-mediated cellular response [161,162,163]. Both devices detect the changes of optical properties caused by mass redistribution within the cells associated with morphology changes external stimuli. However, there are still challenges in integrating these devices with cell microarray formats. Furthermore, all the cells should be cultured in close proximity to the sensor surfaces since these methods are effective only within 200 nm from the surface, which disables the cell-based assay using 3D cellular spheroids. Another label-free assay is electrochemical impedance measurement, which is also called as electric cell-substrate impedance sensing (ECIS) [164,165]. In order to utilize this method for cell microarray, cells are cultured on the array of gold electrodes, the electrical impedance of which is influenced by cell morphology change since the extracellular ionic current pathways are altered by any changes in cell morphology. Electrically excitable cells such as neurons and cardiomyocytes, which can generate different electrical signals with compounds and concentration dependent manners, can be cultured on the microelectrode array and have been also used as label-free assay by recoding the electrical signals [166,167,168]. Because of beating characteristics, cardiomyocyte can be also used as label-free assay by recording the change of beating interval by external stimuli.

### 4.2. Drug Discovery

Cell-based microarray assays have become an essential procedure for drug discovery because these methods are exceptionally useful for evaluating possible drug candidates with vast parallelism. Throughout history, the overall libraries of chemicals and genetic collections have been continuously expanding, which have driven the need for improved cell-based drug discovery screening technology. In the beginning, such experiments were conducted in microtiter plates, which were provided in 96-well and up to 1536-well formats. However, with the introduction of cell microarrays, the situation has improved dramatically, allowing for tens of millions of simultaneous assays in a single step. In addition, with an extremely scaled-down assay, only small amounts of reagents and samples are required; therefore, improved financial characteristics are guaranteed [6,8,169].

For further enhancement of miniaturized devices for a drug screening system, the cell microarray should be integrated with a microfluidic maintenance complex. A microfluidic device consists of a platform patterned with micro-scale channels to ensure a fluidic movement. This combined cell-based biosensor is now becoming a dominant tool for chemical and drug screening. The implementation of microfluidic systems is advantageous over a plain drug screening as mentioned earlier. Although a cell microarray system provides outstanding conditions for drug screening, there is one problem that is worth considering: reproduction of cell-cell interactions observed in living organisms. For in vitro studies, researchers normally deal with homogeneous groups of cell types, but in real living organisms, there are active interactions among different types of tissues. Therefore, toxic reactions in some tissue types could also be affected by metabolic activities of other surrounding tissues, and drug screening of different tissues in isolation will not yield reliable results. This limitation may be resolved by creating a microsystem with interconnected space using a microfluidic system, where each capacity hosts different cell types while the same screening is being conducted overall. Because the novel concept of a “lab-on-a-chip” has become popular in biological research fields these days, this combined system will definitely contribute to the idea. An “organ-on-a-chip” can be accomplished too, allowing for examination of organ-like behaviors on a chip-based system [170,171,172,173].

**Figure 13:**
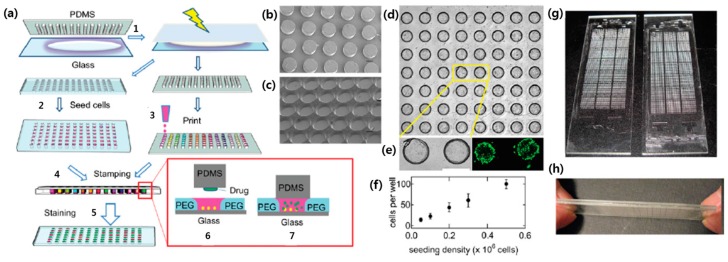
Fabrication of a microarray platform through sandwich system: (**a**) Schematic representation of the fabrication process; (**b**) PDMS posts; (**c**) Microwells; (**d**) Optical microscopy image of the cancer cells seeded within microwells; (**e**) Magnified optic and fluorescent image of the seeded cells; (**f**) Numeric data of seeded cells per well depending on the seeding density; (**g**) A photograph of the PDMS posts (left) and the microwell (right); (**h**) A photograph of the sandwiched system (reproduced with permission from [176]).

For the successful high throughput drug screening system, controlled delivery of different drug candidates into cells microarray can be achieved by various method including drug patterning, stamping and microfluidic loading [174]. Bailey et al. utilized drug patterning method by generating microarray of drug-embedded biodegradable polymer [175]. Cells were seeded onto the microarrays and interacted with drug candidates that were slowly released from biodegradable polymer micropatterns. Researchers could carry out synthetic lethal screen of 70 compounds with A549 or HeLa cells by using a calcein acetoxymethyl ester fluorescence viability assay. The drug screening systems using stamping method have two different microarray chips; one is cell microarray and the other is drug-loaded chip. These two chips were aligned and sandwiched so that each cell spot is exposed to one target drug or drug combination, which consequently prevent crosstalk between neighboring assays. Khademhosseini group developed a sandwich drug screening platform [176]. In their system, cell microarray was prepared with PEG hydrogel microwells and drugs were loaded onto the array of PDMS post. These two chips were sealed together so that each microwell is addressed by a single drug-loaded PDMS post as shown in Figure 13.

**Figure 14:**
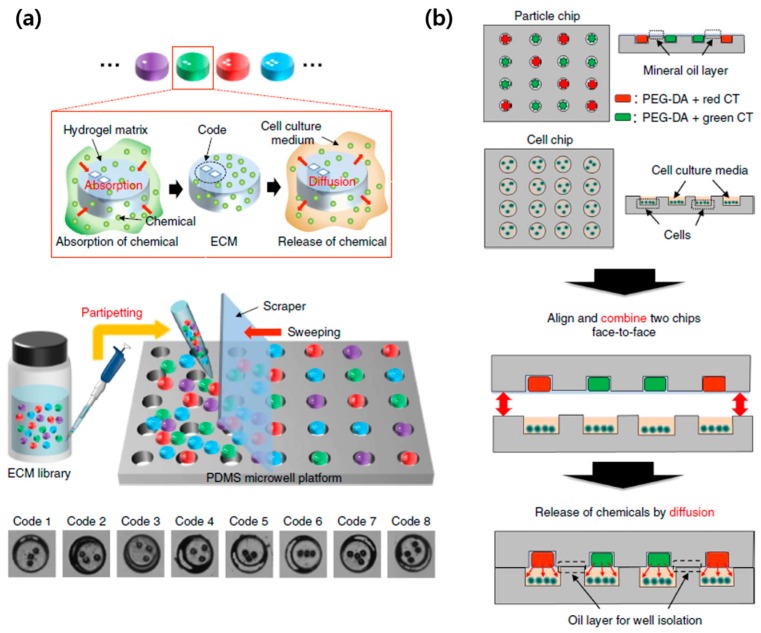
A cell-based microarray system achieved by encoded chemical-laden microparticles (ECMs) and PDMS chip: (**a**) Encoded drug-loaded hydrogel microparticles that are inserted within the PDMS microwell platform; (**b**) Combined system of particle and cell chips enables multiparametric screening of various drugs in different concentration (reproduced with permission from [177]).

The resultant microarray system was able to carry out screening of 320 drug candidates for potential anti-cancer agents by using cytotoxicity assay with MCF-7 human breast cancer cells. Kwon’s group proposed different stamping method by sandwiching particle chip and cell chip [177]. Unique feature of this system is the encoded drug-loaded hydrogel microparticles that were inserted into microwells. The loaded drug was easily identified by the codes on the hydrogel microparticles and released to the cells as shown in Figure 14a.

In this system, multiple drug-embedded microparticles could be loaded into a single microwell in particle chip, enabling the generation of various combination of different types and concentrations of drugs and the investigation of their effects on the cells with high-throughput manner (Figure 14b). Microfluidic system has also been used to deliver the drugs in high-throughput cell-based screening system since different drugs can be independently introduced to cell microarray through different microchannels and a wide range of drug concentration can be generated using passive mixing system [178]. For example, Jarayaman et al. developed microfluidic cell-based microarray integrated with the drug delivery system capable of generating different concentration of two different drugs [179]. Yu et al. also developed microfluidic hepatocyte cell-based microchip where the multiple channels integrated with concentration gradient generator allow the simultaneous administration of different drug candidates or different concentrations of a drug candidate to hepatocytes [180].

### 4.3. Biosensor for Pathogens and Toxins

Conventionally, biosensors for detecting environmental threats like pathogens and toxins are carried out using enzyme, antibody or nucleic acid as probe molecules, which rely on molecular recognition for proper identification and quantification of a particular target. However, those methods provide only analytical information without giving functional information, that is, effects of targets on biological and physiological systems in our body [181,182,183]. Furthermore, those biosensors can be only used for the detection of known target since their detections are based on the specific interaction between probe molecules and target, where one probe molecules are specifically designed or chosen for one target *molecule*. Therefore, conventional biosensors cannot be used for detection of newly-developed chemical and biological warfare weapons.

In the past, living animals have been used as real biosensors when people needed some insights into unknown toxic molecules, because conducting a direct experiment on humans is dangerous. More than one hundred years ago, canaries were used to detect toxic gases in coal mines. There is also a record of US troops using chickens as a means of detection of warfare agents used in wars against Iraq [4,184]. However, because of economic and ethical issues of using animals as sensing elements, there have been many attempts at functional characterization of pathogens and toxicants using cell-based biosensors. Unlike conventional sensing elements such as enzymes or antibodies, cells can respond to various compounds, which enable them to detect unknown targets [185]. For example, if cell viability decreased after exposure to unknown samples, it can provide the information that toxic components may be included within the sample. As mentioned earlier, cell-based biosensors, which incorporate cellular components, represent a distinct enhancement in terms of functional information [186]. For example, in a cell-based biosensor system for pathogen detection, these types of assays not only indicate the presence of pathogens, but they can also provide practical instructions about the pathogens such as mode of action of the pathogen or toxin as well as biological and physiological host–pathogen interactions [187,188].

**Figure 15:**
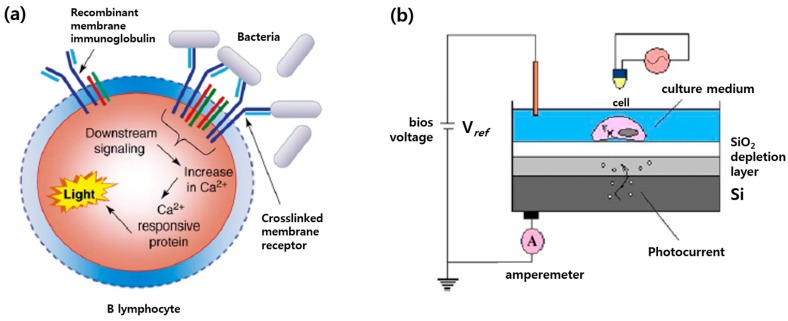
Cell-based detection mechanisms of pathogens and toxins: (**a**) Representation of pathogen detections through cellular membrane receptors; (**b**) Light-addressable potentiometric sensor used to inspect the continuous beatings of the cells (reproduced with permission from [184,193]).

Cell-based detection of pathogens and toxins can be achieved via two different methods [184]. First method is using cellular membrane receptors based on the fact that the interaction between cell and analyte is initiated by the receptor-analyte binding. Rider et al. developed genetically engineered B cell lines, which are capable of detecting pathogens using membrane incorporated pathogen-specific antibodies as shown in Figure 15a [182,184,189]. Binding of pathogens to antibodies induced that make engineered B cells to emit light. The measurement of photon count from emitted cells enabled the quantification of specific pathogens. Virus-specific antibodies were also electro-inserted in the membrane of fibroblast cells and the binding between virus and antibodies were monitored by the principle of the bioelectronic recognition assay using microelectrodes [190]. Secondly, cell-based biosensing can be performed using viability assay that evaluate cytotoxicity of pathogens or toxins. Various cytotoxicity/viability assays described in toxicology sections have been utilized for cell-based assays. For example, exposure of viable cells to pathogens or toxic agent caused the decrease of cell viability, which could be detected optically or electrically. Campbell et al. used ECIS to monitor the infections virus since viral-induced cell death caused impedance changes [191]. B-lymphocyte cells encapsulated in 3D collagen matrix were used for rapid detection of pathogens and toxins by detecting the intracellular enzyme released from damaged cells [192]. The changes in beating frequency, amplitude, and duration of cardiomyocytes were also used for the evaluation of different heavy metal toxicity as shown in Figure 15b [193,194,195], while neural network on microelectrode arrays could be used for the quantification of toxic agents [196,197].

## 5. Conclusions and Future Perspective

Cell-based biosensors constitute a promising field that has numerous applications ranging from pharmaceutical screening to detection of pathogens and toxins. The trends toward miniaturization of cell-based biosensors continue to spur the development of cell microarrays. Although most of the cell microarrays are still being analyzed by means of a positional microarray system, a suspension microarray and integration of a cell microarray with microfluidic devices have received much attention owing to the ease of multiplexing as well as experimental and economic efficiency. Furthermore, cell microarrays are prepared in a biomimetic 3D environment such as a hydrogel and nanofiber to minimize the difference between an animal study and a cell-based assay. Eventually, 3D cell microarray should be combined with microfluidic system, enabling high-throughput screening of bioactive agents for drug discovery as well as detection of pathogens and toxins. However, there are still significant technical challenges to be addressed for cell microarray to extend their applications and be commercialized. First, cell-based sensing has low specificity since various toxic agents result in similar cell damages. Incorporation of probe molecules such as antibodies into cell membrane can enhance the specificity of cell-based assay. Second, cells are prone to be damaged by slight changes of external environments, and therefore, most of cell-based assays are carried out in the laboratory. Using 3D cell culture such as cell-encapsulating hydrogel may provide some protective environments to the cells as well as biomimetic environments and enable the on-the-spot detection of various toxic agents. Unlike proteins and nucleic acids in microarray formats, storing and packaging cell microarray require more complicated technologies such as cryopreservation, which currently reduced cell viability. After those technical issues are addressed, highly integrated cell-based microdevices will find various applications in basic biomedical/pharmaceutical research and form a new market in biosensor-related industries.

## Figures and Tables

**Table 1:** Summary of different advantages and disadvantages between 2D and 3D cellular microarray methodologies.

			Advantages	Disadvantages
**2D**	Positional array	Photolithography	- Well-established, simple and easy procedure - Easiness of controlling the shape and position of cellular patterns by changing the photomask design	- Cellular observation is limited to 2D substrates - Use of photoresists and organic solvent might be cytotoxic
		Soft lithography	- Enhanced producibility with re-usable polymeric stamp - Multi-phenotypic observations are easily possible. - Become highly efficient by incorporation with microfluidic system - Can generate micropatterns on non-planar substrates	- Fabrication process of polymeric stamp or mold is difficult and time-consuming, - Cell patterning is limited to 2D substrates
		DEP	- Cellular pretreatment is unnecessary - High spatial resolution - Easy combinations with other techniques - Can handle a substantial number of living cells at a time	- Additional experimental set-up for generating electric field should be prepared
	Suspension array	Optical-encoding	- Have greater flexibility for multiplex assay - Encoding process is simple - Fluorescence observation is straightforward	- Limited number of color combination - Spectral overlap between coding color and assay color - Photobleaching
		Graphical-encoding	- Unlimited number of coding - Easily decoded without expensive equipment	- Fabrication procedure is more complicated
**3D**	Hydrogel-based		- Provide ECM-mimicking environment for cells - Highly biocompatible - Excellent tunability for various physical properties such as mechanical strength and permeability	- Mechanical strength is relatively weak for long-term support - Gelation process can cause cytotoxicity
	Nanofiber-based		- Provide ECM-mimicking environment for cells - Easily produced at low cost	- Not easy to obtain distinct cellular micropatterns - Low micropattern resolution - Cells may exist only on top of nanofiber matrix
